# Vitamin D on Cardiac Function in Heart Failure: A Systematic Review and Meta-Analysis of 10 RCTs

**DOI:** 10.31083/j.rcm2411325

**Published:** 2023-11-23

**Authors:** Xuemeng Chen, Wenli Zhao, Yan Zhao, Jingchao Ma, Huaien Bu, Ye Zhao

**Affiliations:** ^1^Department of Public Health, International College, Krirk University, 10220 Bangkok, Thailand; ^2^Liver Center, Saga University Hospital, Saga University, 849-8501 Saga, Japan; ^3^School of Health Science and Engineering, Tianjin University of Traditional Chinese Medicine, 300193 Tianjin, China

**Keywords:** vitamin D, heart failure, cardiac function, meta-analysis, randomized controlled trial

## Abstract

**Background:**

Systematic evaluation of the effects of vitamin D supplementation in heart failure (HF) patients.

**Methods:**

Searches were conducted on National Library of Medicine, Web of Science, Cochrane Library, Google Scholar, China National Knowledge Infrastructure, and WANFANG databases. We analyzed data by using Review Manager 5.4 software. All are from the earliest records to March 2023. Outcome indicators analyzed the left ventricular ejection fraction (LVEF), the left ventricular end-diastolic internal diameter (LVEDD), the B-type brain natriuretic peptide (BNP) level and the 25-hydroxy vitamin D (25(OH)D) level.

**Results:**

Ten studies with 1099 patients were included. LVEF (mean difference (MD) = 0.74, 95% CI: –0.29 to 1.76, *p* = 0.41), LVEDD (MD = –0.59, 95% CI: –1.83 to 0.66, *p* = 0.25), BNP (MD = –0.08, 95% CI: –0.24 to 0.08, *p* = 0.34), 25(OH)D (MD = 0.41, 95% CI: –0.28 to 1.11, *p *= 0.25) are not statistically significant. And there is no heterogeneity in the results of LVEF, LVEDD and BNP indicators.

**Conclusions:**

Vitamin D supplementation may not be helpful in the clinical management of patients with HF.

## 1. Introduction

Heart failure (HF) is a potential outcome of end stage of heart disease. It impairs the cardiac circulation due to a systolic and/or diastolic function damage of the heart [1, 2]. HF occurs mainly due to remodeling of heart muscle cells [3]. Vitamin D deficiency triggers excessive activation of the renin-angiotensin-aldosterone system (RAAS) which damages the endothelial function, accelerates the ventricular remodeling, and thus may lead to HF [4]. More than 1 billion people worldwide are deficient in vitamin D leading to the World Health Organization defining it as a public health problem [5]. Vitamin D deficiency is associated with lifestyle risk factors, living conditions, and diseases which usually reduce vitamin D intake, absorption, or synthesis [6]. Low levels of vitamin D may exacerbate chronic HF [7]. However, it has been reported that vitamin D supplementation does not produce long-term benefits in HF patients [8]. Furthermore, a meta-analysis has indicated that vitamin D supplementation does not reduce mortality or improve the left ventricular function [9].

Vitamin D is an essential fat-soluble steroid hormone [10]. Under the ultraviolet B radiation from sunshine, 7-dehydrocholesterol inside skin is converted to vitamin D under the non-enzymatic photolysis [11]. After being released into blood circulation, it is metabolized into 25-hydroxy vitamin D (25(OH)D) by the 25-hydroxylase in the liver [12]. Then 25(OH)D is converted to the active calcitriol by the enzyme 1a-hydroxylase in the kidney [13]. The best method of assessing vitamin D levels in human body is through 25(OH)D [14]. Decreased levels of 1α, 25-dihydroxy vitamin D3, an active form of 25(OH)D in the heart extracellular matrix, influence other forms of vitamin D and contribute to HF progress [15].

Vitamin D plays an important regulatory role in calcium and phosphorus metabolism [16]. It is anti-inflammatory, immunomodulatory, and affects vascular remodeling, blood glucose regulation, reduction of renin, angiotensin, and aldosterone activity in addition to playing a variety of other biological roles [17]. When calcitriol binds to the vitamin D receptor (VDR), its physiological effects are exerted [18]. Because VDR can express in vascular tissues, it may affect calcium in-flow, muscle relaxation, and diastolic function of vascular tissues [19]. As a result, the vitamin D has potential inhibitory effects on cardiac hypertrophy and anti-heart failure [20]. Vitamin D on cardiac function in patients with HF has controversial findings. This study intends to clarify the role of vitamin D in patients with HF.

## 2. Methods

### 2.1 Search Strategy

Searches were conducted on National Library of Medicine, Web of Science, Cochrane Library, Google Scholar, China National Knowledge Infrastructure, and WANFANG databases. All are from the earliest records to March 2023. Searching terms included “heart failure”, “vitamin D”, “Cardiac failure”, “Randomized Controlled Trials”, “Vitamin D3” and “cardiac function”. The languages of the literature were mainly English and Chinese. The relevant literature was traced in the references of the retrieved clinical trial report papers or reviews. Protocol registration prior to initiating the meta-analysis was not possible due to missing a selective time point in the design period.

### 2.2 Inclusion Criteria

We include randomized controlled trials (RCT) of vitamin D in patients with HF. The definition of HF is based on the New York Heart Association (NYHA) classification ≥II or the left ventricular ejection fraction (LVEF) ≤40%. Study subjects are supplemented with only vitamin D as the micronutrient. The treatment group adds vitamin D and the control group uses placebo or no drug, both under the maintenance of a usual treatment.

### 2.3 Exclusion Criteria

The followings are excluded: (1) trials reported in abstract only, (2) low-quality literature, (3) cohort studies, (4) retrospective case-control studies, (5) conference literature, (6) repetitive articles, (7) and nonclinical trials.

### 2.4 Literature Quality Assessment

Evaluators first independently completed the initial screening of the included literature by reading the title and abstract. The methodological criteria of quality assessment are based on Cochrane and meet the inclusion criteria. By reading some full texts, the following evaluation criteria were used: (1) randomization, (2) concealment of allocation, (3) subject and intervention blinding, (4) blinding on outcome assessments, (5) data integrity, (6) selective outcome reporting, and (7) other biases. “Uncertain risk”, “low risk”, and “high risk” evaluations were used for the assessment of bias. Two independent researchers evaluated data quality. One third party was solicited to advice when discussion could not resolve the inconsistent opinion of a particular study’s inclusion.

### 2.5 Data Extraction

The data extracted includes the following: (1) general information, such as title, author, year of publication and trial quality score, (2) comparability of data and interventions across patient data groups, and (3) outcome data including 25(OH)D, LVEF, left ventricular end-diastolic internal diameter (LVEDD), and B-type brain natriuretic peptide (BNP).

### 2.6 Statistical Analysis

Statistical analysis was performed using Review Manager 5.4 software (International Cochrane Collaboration Network, TX, USA), with a test level of α = 0.05. Continuous variables were analyzed by using the mean difference (MD) and 95% confidence intervals (CI). Clinical heterogeneity of the included studies was first analyzed, followed by statistical heterogeneity using the *$I^{2}$* test [21]. When *p* > 0.1 and *$I^{2}$* < 50%, homogeneity among several similar studies can be considered and a fixed effect model is used to analyze. When *p* <0.1 and *$I^{2}$* > 50%, heterogeneity is considered and a random effect model is used to analysis. *$I^{2}$* > 50% indicates high heterogeneity, *$I^{2}$* of 25%–50% reveals moderate heterogeneity, and *$I^{2}$* < 50% shows low heterogeneity [22]. If heterogeneity was found, the source was analyzed followed by a sensitivity analysis.

## 3. Results

### 3.1 Characteristics of Study and the Quality

The basic characteristics of the included studies are shown in Table 1 (Ref. [23, 24, 25, 26, 27, 28, 29, 30, 31, 32]). In all included studies, 25(OH)D is an outcome indicator, and the most timeframes are from 3 months to 4 months. The study’s quality evaluation is indicated in Table 2 (Ref. [23, 24, 25, 26, 27, 28, 29, 30, 31, 32]). Most studies have low risk for all items, so the included studies are quality.

**Table 1.** **Basic characteristics of the included studies**.

Author	Experimental design	Vitamin D supplementation dose	Periodicity	Test population	Key outcome indicators
Qu *et al*. [23], 2015	Forward looking	1000 U/d	3 months	Ischemic heart failure; NYHA classification III–IV	25(OH)D; BNP; LVEF
Li *et al*. [24], 2015	Forward looking	1000 U/d	3 months	Children with chronic heart failure; 3 years < age >1 month	25(OH)D; NYHA classification; Cardiac efficacy
Wu *et al*. [25], 2011	Forward looking	1600 U/d	10 weeks	Chronic heart failure; NYHA classification ≥ II; 25(OH)D < nmol/L	25(OH)D; BNP; NYHA classification; 6-minute walking distance (6MWD)
Nicolas [26], 2013	Forward looking	2000 U/d	6 weeks	Chronic heart failure; age ≥18 years; LVEF <45%; NYHA classification II	25(OH)D; NYHA classification; 6MWD
Zittermann *et al*. [27], 2019	Forward looking	4000 U/d	12 months	Advanced heart failure; 25(OH)D <75 nmol/L; 18 years < age >79 years; NYHA classification ≥ II	25(OH)D; LVEF
Soad *et al*. [28], 2012	Forward looking	1000 U/d	3 months	Infants with ischaemic heart failure; EF <40%	25(OH)D; LVEF; RAS cytokines
Klaus *et al*. [29], 2016	Forward looking	4000 U/d	12 months	Chronic heart failure; LVEF ≤45%; 25(OH)D <50 nmol/L; NYHA classification II–III	25(OH)D; LVEF; 6MWD
Rebecca *et al*. [30], 2014	Forward looking	50,000 U/w	6 months	Heart failure; age ≥50 years; 25(OH)D ≤37.5 ng/mL; NYHA classification II–IV	25(OH)D; PTH
Woo* et al*. [31], 2022	Forward looking	4000 U/d	4 months	Chronic heart failure; 25(OH)D <75nmol/L; NYHA classification II–III	25(OH)D; LVEF; NYHA classification; 6MWD
Heidi [32], 2017	Forward looking	10,000 U/d	6 months	Heart failure; NYHA classification II–III; age ≥18 years; 25(OH)D ≥32 ng/mL	25(OH)D; BNP; QOL; CPX; PTH

Note: 25(OH)D, 25-hydroxy vitamin D; BNP, B-type brain natriuretic peptide; LVEF, left ventricular ejection fraction; NYHA, New York Heart Association; 6MWD, 6-minute walking distance; RAS, renin-angiotensin; PTH, parathyroid hormone; QOL, quality of life; CPX, complete physical examination.

**Table 2.** **Study quality evaluation**.

Included studies	Radom allocation	Allocation concealment	Double blind method	Evaluation of blindness	Data integrity	Selective report	Others
Qu *et al*. [23], 2015	Unclear	Low risk	Low risk	Unclear	Low risk	Low risk	Low risk
Li *et al*. [24], 2015	Unclear	Low risk	High risk	Unclear	Low risk	Unclear	Unclear
Wu *et al*. [25], 2011	Unclear	Low risk	Low risk	Unclear	Low risk	Unclear	Unclear
Nicolas [26], 2013	Low risk	Unclear	Low risk	Low risk	Low risk	Low risk	Low risk
Zittermann *et al*. [27], 2019	Unclear	Unclear	Unclear	Unclear	Low risk	Unclear	Low risk
Soad *et al*. [28], 2012	Unclear	Unclear	Low risk	Low risk	Low risk	Low risk	Unclear
Klaus *et al*. [29], 2016	Low risk	Unclear	Unclear	Low risk	Low risk	Unclear	Low risk
Rebecca *et al*. [30], 2014	Low risk	Low risk	Low risk	Unclear	Low risk	Unclear	Low risk
Woo* et al*. [31], 2022	Low risk	Unclear	Low risk	Unclear	Low risk	Unclear	Unclear
Heidi [32], 2017	Low risk	Low risk	Low risk	Unclear	Low risk	Unclear	Low risk

Unclear, Not specified in the article; Low risk, There are specific instructions in the article; High risk, Not mentioned in the article.

### 3.2 General Information on the Inclusion of Studies

**Fig. 1.**
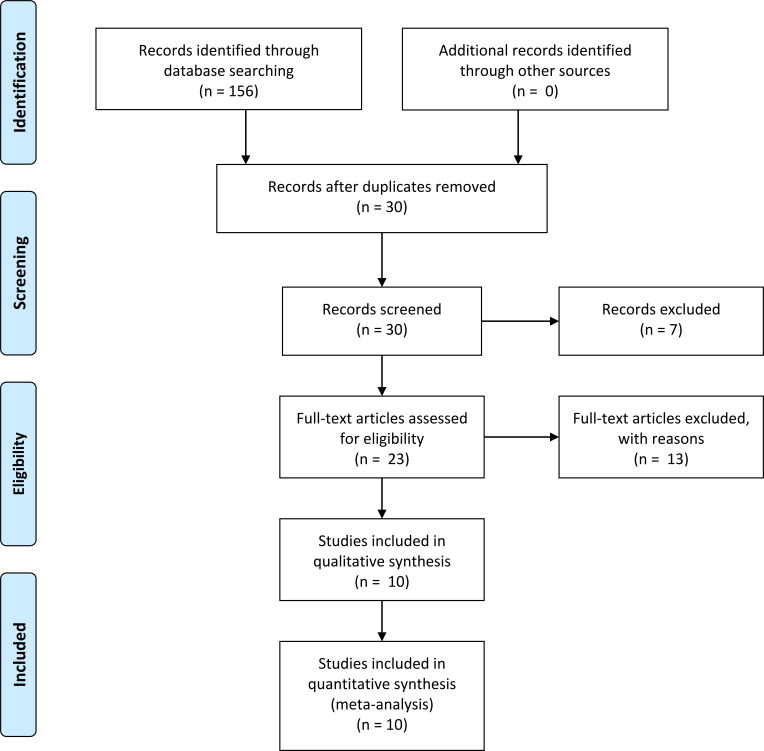
**PRISMA 2009 Flow Diagram**.

**Fig. 2.**
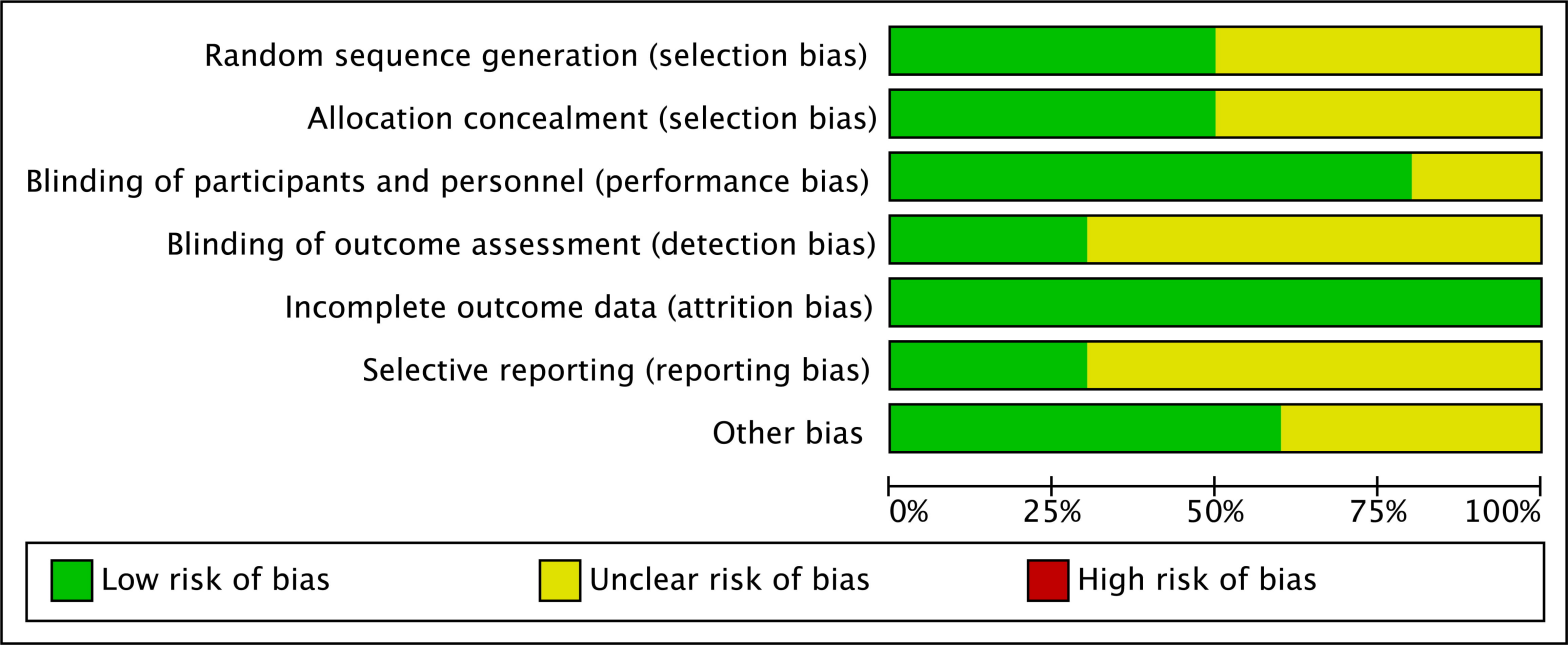
**Bias of studies**.

In total, 10 prior studies met the eligibility criteria and were included [23, 24, 25, 26, 27, 28, 29, 30, 31, 32]. The selection process is described in Fig. 1. There are a total of 1099 patients, with 548 in the vitamin D group and 551 in the control group. Overall, 5 studies [26, 29, 30, 31, 32] refer to the correct randomization method and 5 studies [23, 24, 25, 30, 32] adopt allocation concealment; 4 studies [26, 29, 31, 32] report LVEF, 4 studies [27, 28, 29, 31] report LVEDD, 4 studies [23, 25, 27, 32] report BNP, 7 studies [24, 27, 28, 29, 30, 31, 32] report 25(OH)D and 1 study [24] report the occurrence of adverse events during treatment . The bias of the study is analyzed in Fig. 2.

### 3.3 Vitamin D Effects on Cardiac Function

#### 3.3.1 LVEF

**Fig. 3.**
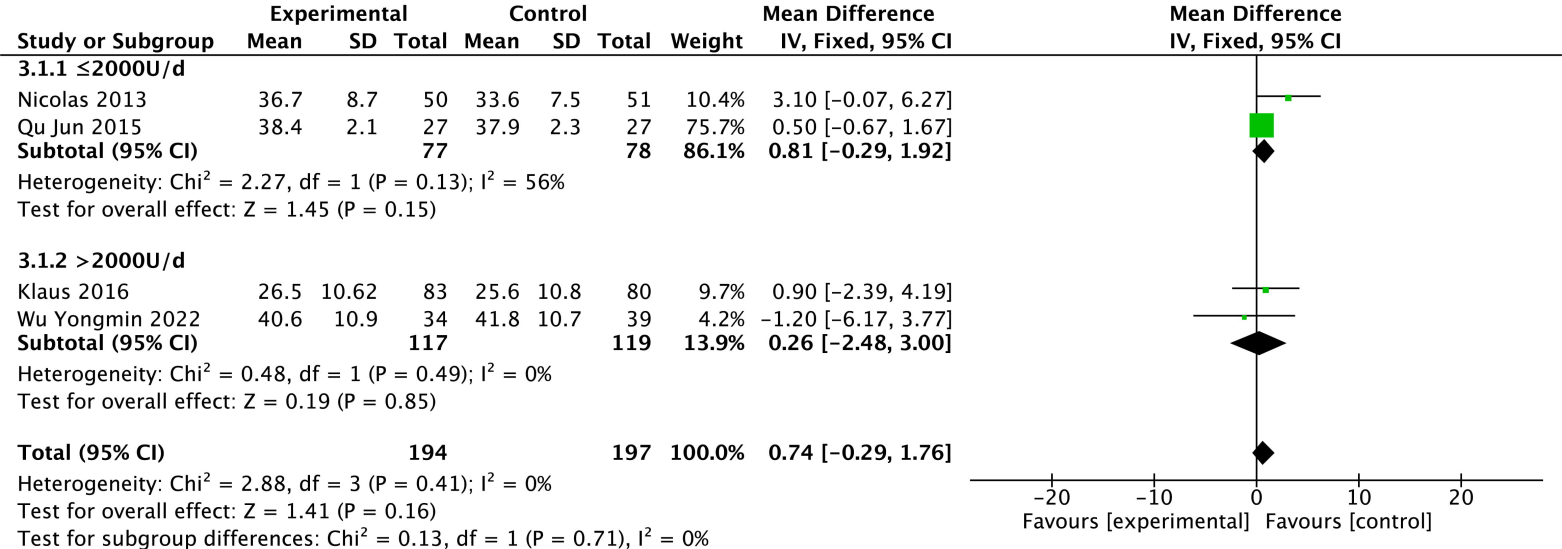
**Changes in LVEF after treatment in the vitamin D and control groups**. LVEF, left ventricular ejection fraction.

Levels of LVEF were reported in four studies [26, 29, 31, 32]. There is no heterogeneity in the results among studies (*p* = 0.41, *$I^{2}$* = 0%) with the use of a fixed effect model. Based on the overall-effect test, there is no statistically significant difference in LVEF between the two groups (Z = 1.41, *p* = 0.16). There is no significant difference in LVEF between the vitamin D group and the control group (MD = 0.74, 95% CI: –0.29 to 1.76). According to the vitamin D usage subgroup analysis, there is no statistically significant difference between the two groups for doses <2000 U/d (*p* = 0.15) or for doses >2000 U/d (*p* = 0.85). The results of the different subgroups according to the different doses show that LVEF is not statistically significant between the test and control groups. The results are shown in Fig. 3.

#### 3.3.2 LVEDD

**Fig. 4.**
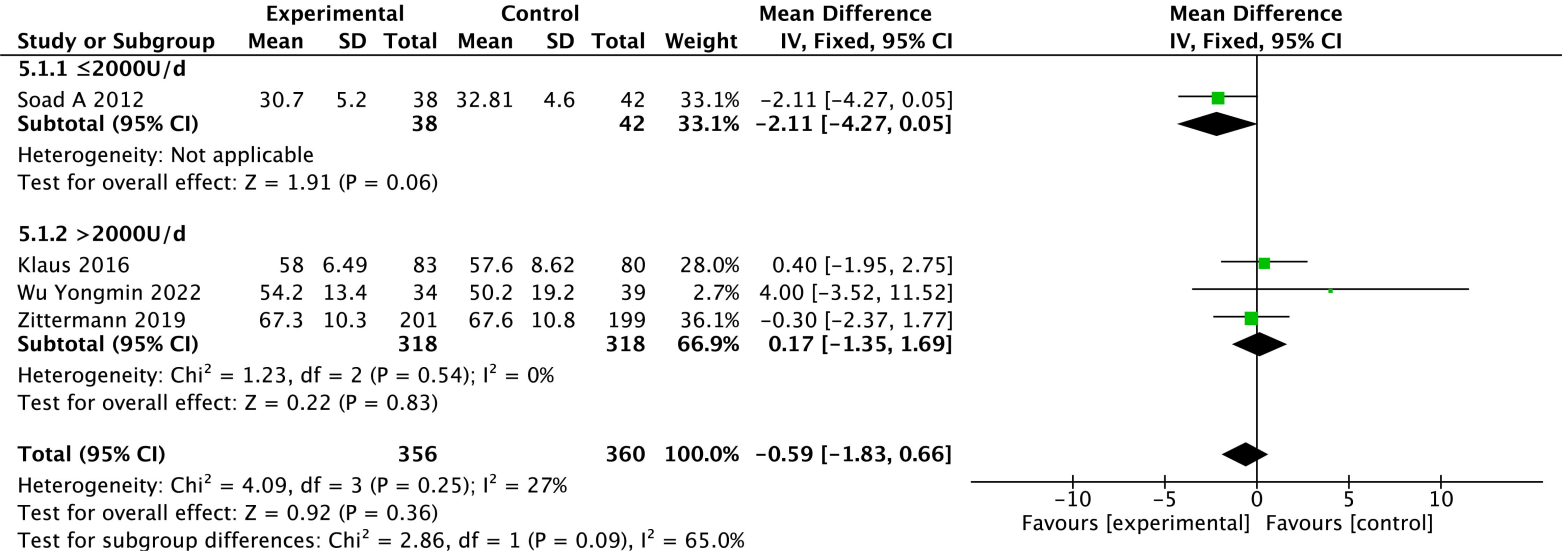
**Changes in left ventricular end-diastolic internal diameter after treatment in the vitamin D and control groups**.

Levels of LVEDD were reported in four studies [27, 28, 29, 31]. There is no heterogeneity in the results among studies (*p *= 0.25, *$I^{2}$* = 27%) with the use of a fixed effect model. Based on the overall-effect test, there is no statistically significant difference in LVEDD between the two groups (Z = 0.92, *p* = 0.36). There is no significant difference in LVEDD between the vitamin D group and the control group (MD = –0.59, 95% CI: –1.83 to 0.66). According to the vitamin D usage subgroup analysis, there is no statistically significant difference between the two groups for doses <2000 U/d (*p* = 0.06) or for doses >2000 U/d (*p* = 0.83). The results of the different subgroups according to the different doses show that LVEDD is not statistically significant between the test and control groups. The results are shown in Fig. 4.

#### 3.3.3 BNP

**Fig. 5.**
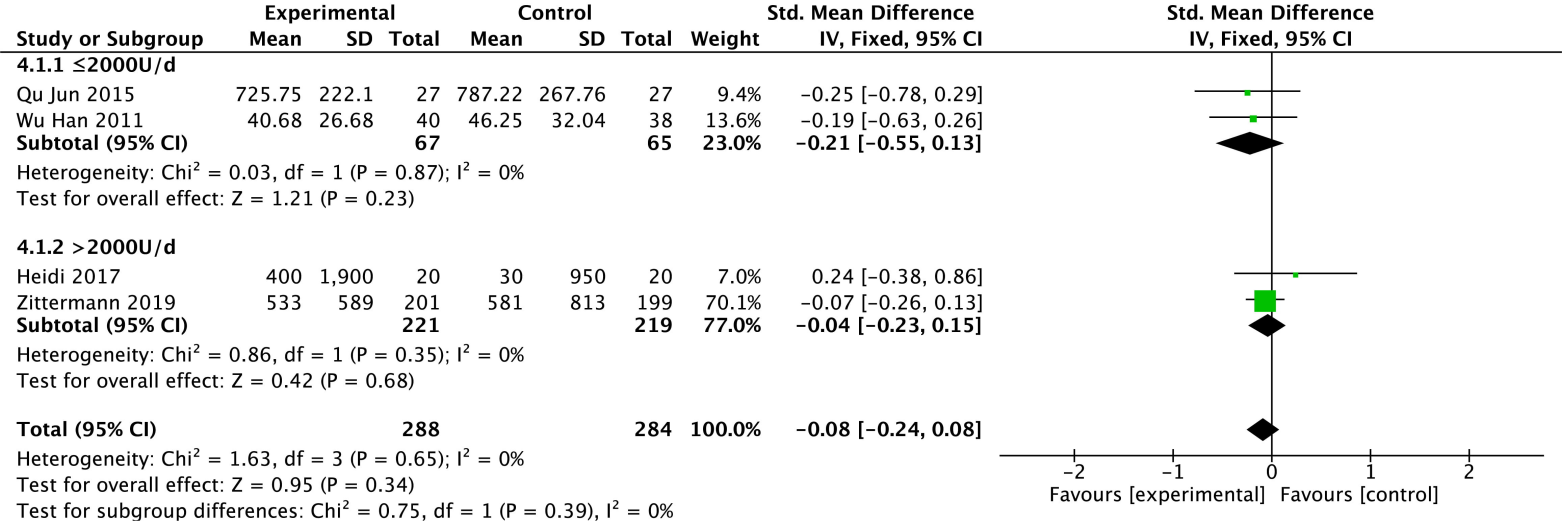
**Changes in B-type brain natriuretic peptide after treatment in the vitamin D and control groups**.

Levels of BNP were reported in four studies [23, 25, 27, 32]. There is no heterogeneity in the results among studies (*p* = 0.65, *$I^{2}$* = 0%) with the use of a fixed effect model. Based on the overall-effect test, there is no statistically significant difference in BNP between the two groups (Z = 0.95, *p* = 0.34). There is no significant difference in BNP between the vitamin D group and the control group (MD = –0.08, 95% CI: –0.24 to 0.08). According to the vitamin D usage the subgroup analysis, there is no statistically significant difference between the two groups for doses <2000 U/d (*p* = 0.23) or for doses >2000 U/d (*p* = 0.68). The results of the different subgroups according to the different doses show that BNP is not statistically significant between the test and control groups. The results are shown in Fig. 5.

#### 3.3.4 25(OH)D

**Fig. 6.**
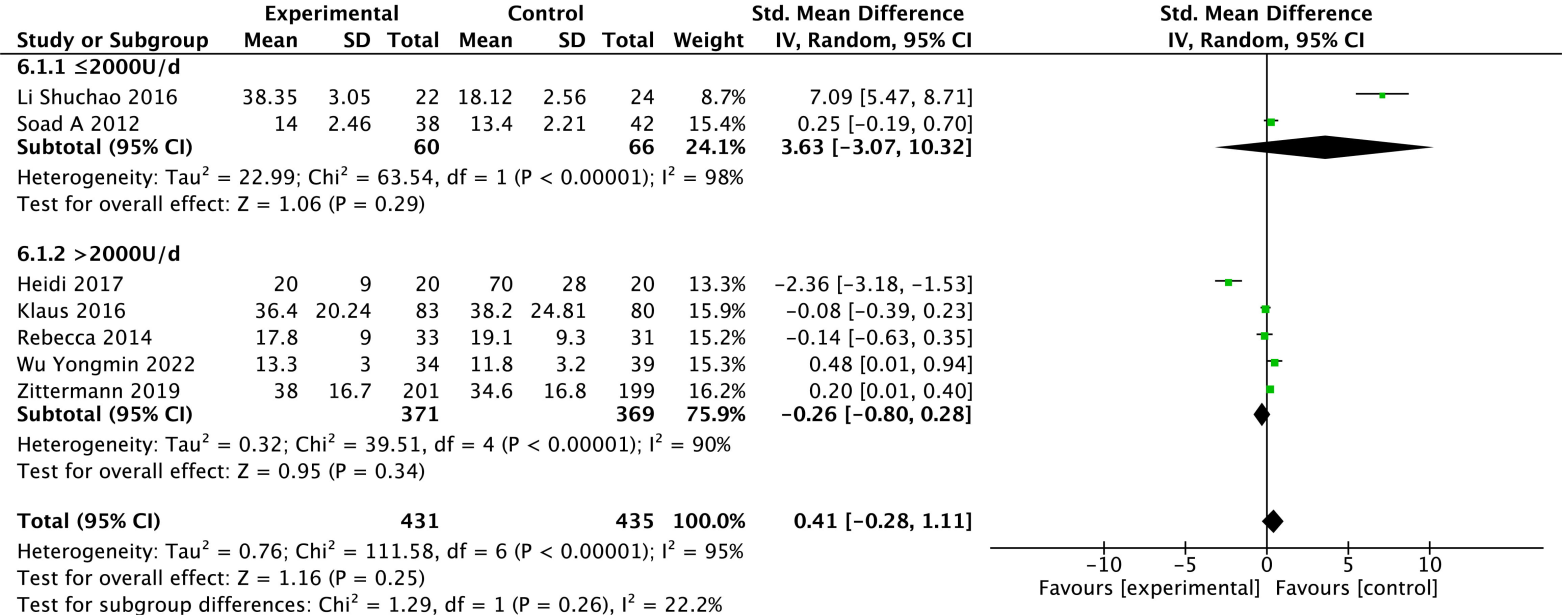
**Changes in 25-hydroxy vitamin D after treatment in the vitamin D and control groups**.

Levels of 25(OH)D were reported in seven studies [24, 27, 28, 29, 30, 31, 32]. Because heterogeneity was found in the study results (*p* < 0.00001, *$I^{2}$* = 95%), a random effect model is used. Based on the overall-effect test, there is no statistically significant difference in 25(OH)D between the two groups (Z = 1.16, *p* = 0.25). There is no significant difference in 25(OH)D between the vitamin D group and the control group (MD = 0.41, 95% CI: –0.28 to 1.11). According to the vitamin D usage subgroup analysis, there is no statistically significant difference between the two groups for doses <2000 U/d (*p* = 0.29) or for doses >2000 U/d (*p* = 0.34). The results of the different subgroups according to the different doses show that 25(OH)D is not statistically significant between the test and control groups. The results are shown in Fig. 6.

### 3.4 Adverse Events

Adverse events (AEs) were only reported in a single study [24]. The most frequent AEs include panic, nausea, dizziness, and fatigue. However, the incidence of adverse events between the two groups is not statistically significant.

## 4. Discussion

Approximately ninety percent of people with chronic HF are vitamin D deficient and low levels of vitamin D are known to activate the RAAS system, triggering the inflammatory response, and leading to endothelial dysfunction [33]. This model predicts a correlation between deficiency of vitamin D and poor prognosis in chronic HF patients, suggesting that vitamin D supplementation may improve left ventricular remodeling and have a role in the recovery of cardiac function [34]. Vitamin D deficiency has been shown to result in cardiovascular complications, while a normal level may have protective effects in ventricular muscle [35]. It is notable that patients failing to complete the trial were excluded from the analysis, thus clinical events for this subgroup were not assessed. The results from this meta-analysis are in agreement with another study that found vitamin D supplementation resulted in no significant change to cardiac structure, systolic function or diastolic function, although the bioactive metabolite 25(OH)D, a nuclear hormone receptor ligand, has anti-hypertrophic activity [36].

The included studies did show an increase of 25(OH)D in the group of vitamin D compared to the control group, and the increase of 25(OH)D was accompanied with increased calcium concentrations in plasma. In one meta-analysis, it is shown that increased calcium concentrations are the feature of HF [37]. HF patients with vitamin D deficiency (25(OH)D <25.0 nmol/L) have a higher mortality rate than those with 25(OH)D >75.0 nmol/L (corrected heart rate 1.61 [95% CI: 1.08 to 2.41]) [38]. We can therefore infer that an increase of 25(OH)D may raise the incidence of HF.

The results of this study show that LVEF, LVEDD, BNP, and 25(OH)D are not statistically significant, nor is there any significant effect on vitamin D supplementation in BNP. There was no heterogeneity in the results of LVEF, LVEDD and BNP indicators, suggesting that vitamin D supplementation is not significantly correlated with left ventricular remodeling. It suggests that vitamin D supplementation is not helpful to treat HF.

A RCT shows that moderately high doses of cholecalciferol adversely affected HF patients [39]. However, the recommended frequency and dose of vitamin D supplementation are not clear. Our subgroup analysis suggests that none of the measured doses of vitamin D supplementation improve the cardiac function of HF patients.

In recent years a number of RCTs have been conducted on the effects of vitamin D in HF patients, but different studies report controversial results. A meta-analysis shows that low vitamin D levels may associate with increased risks of all-cause mortality [40]. Another meta-analysis reported that supplementation of vitamin D did not improve LVEF or mortality in chronic HF [41]. While RCTs provide basis for clinical evidence, trials are often conducted in highly controlled settings with narrow inclusion and exclusion criteria, which can also reduce their generalizability and external validity [42].

This study has some limitations: (1) the included population number is small, (2) the presence of heterogeneity, particularly in blinding methods. It may also be related to differences in vitamin D doses and study populations, (3) the different dose cycles of vitamin D in different trials may also affect the results of the study, and (4) we did not include patients with preserved ejection fraction.

## 5. Conclusions

Since there is no advantage on the LVEF, LVEDD, BNP and 25(OH)D, Vitamin D supplementation may not be helpful in the clinical management of patients with HF.

## Data Availability

The data used to support the findings of this study are included within the article.

## Abbreviations

HF, heart failure; RASS, renin-angiotensin system; 25(OH)D, 25-hydroxy vitamin D; VDR, vitamin D receptor; RCT, randomized controlled trial; LVEF, left ventricular ejection fraction; LVEDD, left ventricular end-diastolic internal diameter; BNP, B-type brain natriuretic peptide; MD, mean difference; CI, confidence intervals.

## Supplementary Material

Supplementary material associated with this article can be found, in the online version, at https://doi.org/10.31083/j.rcm2411325.
